# Lung cavitation to pneumothorax: A case report of the multilayered respiratory challenges in oncology patients

**DOI:** 10.1016/j.ijscr.2023.109157

**Published:** 2023-12-15

**Authors:** Nathaniel Grabill, Mena Louis, Cindy Idowu, Clifton Hastings, Hardeep Singh

**Affiliations:** a Northeast Georgia Medical Center, General Surgery GME Program, United States of America; b Northeast Georgia Medical Center, Cardiovascular and Thoracic Surgery, United States of America; c Northeast Georgia Medical Center, Graduate Medical Education, Research Department, United States of America

**Keywords:** Leiomyosarcoma, Regorafenib, *Pseudomonas aeruginosa*, Pneumothorax, Sepsis, Multidisciplinary management, Community-acquired pneumonia, Lung cavitation, Chemotherapy-induced complications, Thoracotomy, Empyema differential, Surgical risk management

## Abstract

**Introduction:**

The management of patients with complex oncological histories poses unique challenges, particularly when they are on targeted chemotherapy agents known for specific side effects. This case report illuminates the multifaceted complexities encountered in such scenarios, with a focus on the rare complications associated with targeted therapies.

**Case presentation:**

We present a 50-year-old male with an extensive oncological background, including childhood retinoblastoma and radiation-induced leiomyosarcoma. Recently diagnosed with skull base osteosarcoma, he was undergoing treatment with Regorafenib. Admitted with sepsis due to *Pseudomonas aeruginosa*-induced community-acquired pneumonia, his clinical course was complicated by lung cavitation leading to a spontaneous pneumothorax. This report highlights the absence of empyema, a crucial differential in the diagnosis.

**Discussion:**

This case unravels the intricate interplay between targeted chemotherapy, concurrent medications like prednisone, and their potential to cause severe complications such as pneumonia and pneumothorax. It delves into the mechanisms by which Regorafenib can lead to lung cavitation and abscess formation, a rare but significant risk. The importance of a multidisciplinary approach for prompt diagnosis and treatment, including surgical intervention, is highlighted. The pathology of the surgically resected lobe revealed metastatic high-grade leiomyosarcoma, adding another layer of complexity to the case.

**Conclusion:**

This case serves as a cautionary tale highlighting the need for vigilant monitoring of patients on targeted chemotherapy agents, especially those with complex medical histories. It highlights the importance of considering potential drug-related complications and the rationale behind therapeutic choices, including antibiotic selection and surgical decision-making, in the management of acute medical conditions in these patients.

## Highlights

• Complex oncological history with childhood retinoblastoma, radiation-induced leiomyosarcoma, and skull base osteosarcoma.
• The patient presented with sepsis and community-acquired pneumonia while on chemotherapy and prednisone.
• Spontaneous pneumothorax required urgent surgical intervention, revealing metastatic leiomyosarcoma in the resected lobe.
• The case illustrates the potential for cavitation and subsequent complications with targeted chemotherapy agents.
• Emphasizes the need for a multidisciplinary approach and careful medication monitoring.

## 1 Introduction

The management of patients with complex oncological histories often presents clinicians with a range of diagnostic and therapeutic challenges [1,2]. The advent of targeted chemotherapy agents has revolutionized cancer treatment but also introduced new complications, some of which may be severe and necessitate urgent intervention [3,4]. Concurrently, the use of other medications like corticosteroids can also complicate the clinical picture by increasing susceptibility to infections [5,6]. This case report discusses the unique challenges in managing a patient with a complicated oncological history, including childhood retinoblastoma, radiation-induced leiomyosarcoma, and osteosarcoma, who was on targeted chemotherapy and also presented with acute respiratory complications [3,7,8]. It aims to shed light on the potential complications associated with targeted chemotherapy agents, particularly the rare occurrence of lung cavitation leading to severe respiratory complications, and the role of multidisciplinary management in such intricate cases [9].

## 2 Case presentation

### 2.1 Patient background and presentation

A 50-year-old male with a multifaceted medical history was diagnosed with retinoblastoma in childhood, which led to blindness following surgical intervention. Later in life, he developed radiation-induced leiomyosarcoma of the maxillary sinus, initially treated with resection and adjuvant proton therapy. The disease later metastasized, and he was also diagnosed with osteosarcoma of the skull base. The patient has been on various treatments, including Temozolomide and, more recently, Regorafenib, due to its efficacy against both leiomyosarcoma and osteosarcoma. This report highlights the absence of empyema, a crucial differential in the diagnosis.

### 2.2 Clinical presentation

**Fig. 1:**
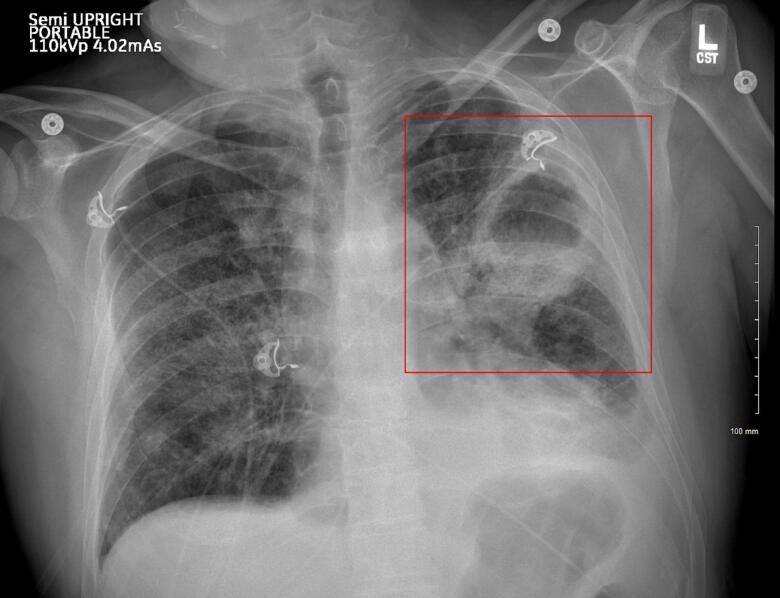
Chest X ray demonstrates a cavitary mass within the left upper lung with increasing thickness seen surrounding the mass. There is increased interstitial density and airspace consolidation seen bilaterally.

**Fig. 2:**
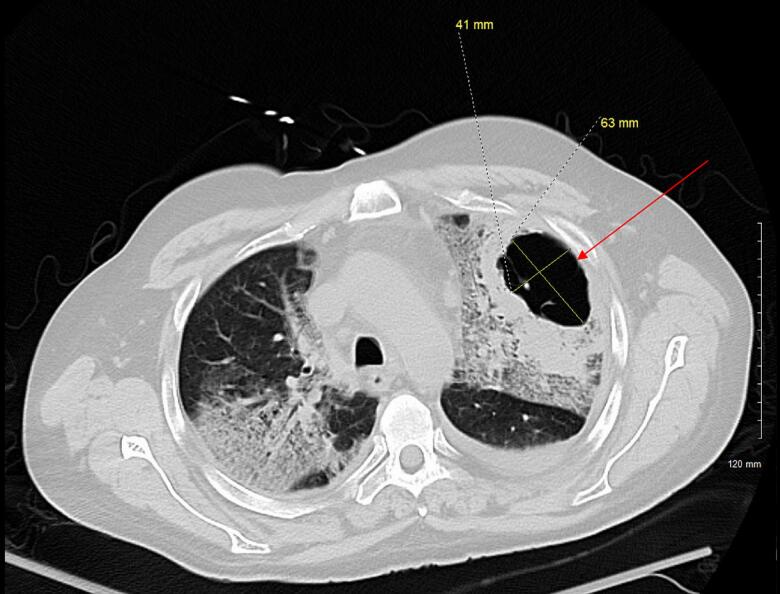
There is a cavitary lesion in the left upper lobe (red arrow) with minimal internal septation that measures 6.3 × 4.1 cm. (For interpretation of the references to colour in this figure legend, the reader is referred to the web version of this article.)

**Fig. 3:**
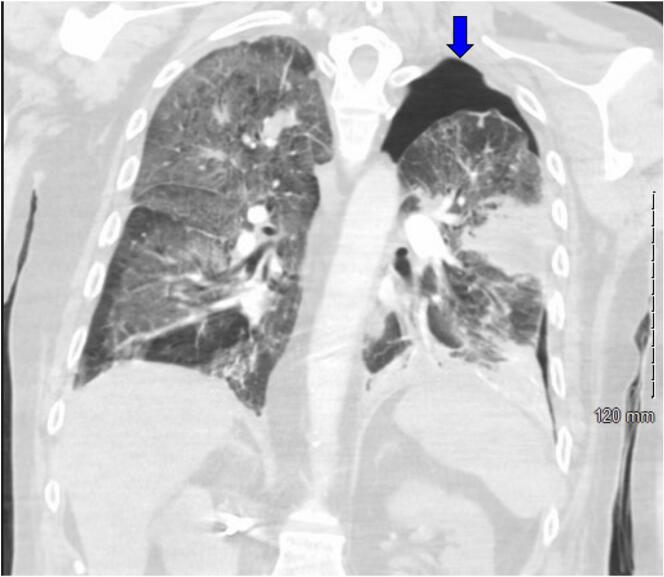
Large left-sided pneumothorax (blue arrow). A left upper lobe cavitary mass within the direct pleural extension and a pleural defect. This is probably the source of the pneumothorax. (For interpretation of the references to colour in this figure legend, the reader is referred to the web version of this article.)

The patient was admitted to the hospital with signs of sepsis—hypotension, leukocytosis, hypoxia, and lactic acidosis secondary to community-acquired pneumonia caused by *Pseudomonas aeruginosa*. The initial chest X-ray showed a left upper lobe cavitary mass (Fig. 1, Fig. 2). His respiratory condition deteriorated rapidly, requiring intubation and mechanical ventilation. On hospital day 4, imaging revealed a left-sided spontaneous pneumothorax necessitating the placement of a chest tube (Fig. 3). The development of lung cavitation, a known but rare complication of Regorafenib treatment, escalated into a spontaneous pneumothorax. Importantly, pleural fluid analysis and clinical findings confirmed the absence of empyema, a crucial differential in this case.

The patient was also on prednisone, raising questions about its role in predisposing him to bacterial infection. His Regorafenib treatment may have contributed to lung cavitation and subsequent pneumothorax, warranting further study on the adverse effects of this targeted therapy.

### 2.3 Diagnostic findings

Initial laboratory tests showed leukocytosis and lactic acidosis. Respiratory cultures confirmed the presence of *Pseudomonas aeruginosa*. A CT scan revealed multiple stable and slightly enlarging nodules, the largest in the right upper lobe. A PET scan also indicated increased metabolic activity in known osseous metastases.

### 2.4 Therapeutic interventions

**Fig. 4:**
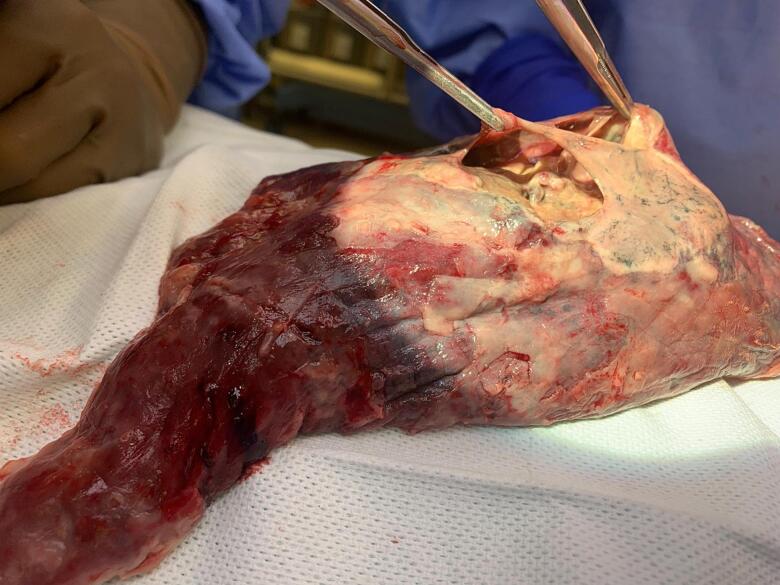
Intraoperative image that displays the resected lobe, which features a necrotic cavity held by 2 forceps.

The patient's initial treatment involved intravenous antibiotics, with vancomycin and amoxicillin-sulbactam, which were later changed to cefepime. A sudden pneumothorax occurred, likely resulting from a ruptured cavity in the left lung. Immediate intervention included the insertion of a chest tube and the acquisition of a CT scan. Persistent air leakage from the chest tube led to consultation with thoracic surgery, culminating in a left thoracotomy, and left upper lobectomy (Fig. 4). In the post-surgical phase, the patient received intravenous meropenem and was successfully extubated on the second postoperative day.

The pathology of the resected lobe showed metastatic high-grade leiomyosarcoma. This was characterized by compact fascicles of spindle cells with cigar-shaped nuclei and eosinophilic cytoplasm, displaying nuclear pleomorphism, frequent mitotic figures, and areas of tumor necrosis. The diagnosis of leiomyosarcoma was further supported by positive staining for Desmin and the absence of staining for other markers (SOX10, HMB45, CK7, CD34, S100).

### 2.5 Follow-up

**Fig. 5:**
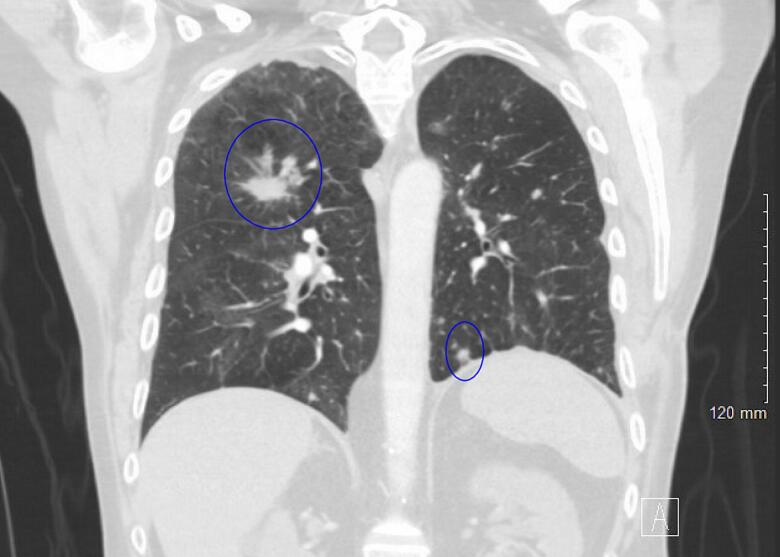
CT chest 2 months postoperatively with complete left lung expansion and stable bilateral nodules. (blue circles). (For interpretation of the references to colour in this figure legend, the reader is referred to the web version of this article.)

He was discharged on a 4-week course of oral ciprofloxacin and home oxygen. Outpatient follow-up was scheduled with both cardiothoracic surgery and pulmonology. At his two-month clinic follow-up, the patient's chest X-ray revealed full lung expansion and stable bilateral lung nodules (Fig. 5, blue circles). He reported feeling well and had no complaints.

## 3 Discussion

Managing patients with multiple oncological comorbidities can be a clinical enigma, requiring a meticulously crafted, evidence-based approach [2,10]. This is particularly evident in our patient, whose history spans childhood retinoblastoma, subsequent radiation-induced leiomyosarcoma, and most recently, skull base osteosarcoma [3]. Each layer of his medical history brings its own set of challenges and risks, complicating the diagnostic and treatment paradigms [1,2].

The patient's treatment with Regorafenib was further complicated by the development of lung cavitation [1,4]. Although Regorafenib is a promising agent for treating various types of cancer, it is not without side effects [1,4,11]. One of the rare but significant complications associated with Regorafenib is lung cavitation, which became a critical issue for our patient [1,11]. The cavitation escalated into a spontaneous pneumothorax, requiring immediate surgical intervention [7,12]. This aspect of the case serves as a crucial reminder of the need for ongoing clinical and radiological surveillance when using targeted chemotherapy agents [7,12].

Adding complexity to the case, the patient was already on prednisone therapy, likely predisposing him to infection. He developed sepsis from *Pseudomonas aeruginosa* pneumonia, a serious bacterial infection that presented another layer of diagnostic and therapeutic complexity [5]. Prednisone, a corticosteroid often used for its anti-inflammatory properties, could have suppressed the patient's immune system, making him more susceptible to this opportunistic infection [5,6].

Postoperative care was also fraught with challenges. Antibiotic therapy had to be adjusted based on the patient's bacterial resistance profile, illustrating the need for an adaptable and dynamic treatment plan. Although immediate postoperative outcomes were favorable, the patient's complicated history mandates ongoing specialized follow-up to effectively manage any future complications or recurrences.

This case exemplifies the intricate challenges and complexities in treating patients with multiple oncological histories, particularly when introducing targeted chemotherapy agents like Regorafenib [1,8]. The rare but significant occurrence of lung cavitation leading to spontaneous pneumothorax necessitated immediate surgical intervention, while the patient's concurrent use of prednisone likely exacerbated his susceptibility to *Pseudomonas aeruginosa* pneumonia [5,6]. The case serves as an educational landmark for clinicians, emphasizing the importance of vigilant monitoring and adaptability in treatment plans, particularly for those with intricate medical backgrounds.

### 3.1 The surgical decision-making process

The decision to proceed with lobectomy in the context of active infection and the patient's immunocompromised status was not taken lightly. The selection of vancomycin and amoxicillin-sulbactam, followed by cefepime, was based on the patient's specific bacterial resistance profile and the urgent need to address his acute respiratory distress. In retrospect, the use of carbapenems could have been considered from the outset, given the patient's immunocompromised state and the aggressive nature of his infection.

### 3.2 Managing surgical risks associated with anti-angiogenic therapy

The risks of delayed wound healing and latent postoperative bleeding associated with Regorafenib, an anti-angiogenic therapy targeting VEGFRs, Tie-2, PDGFR, etc., were significant concerns. Detailed preoperative planning included strategies to mitigate these risks, such as careful monitoring of the patient's nutritional status, optimizing perioperative blood pressure control, and employing meticulous surgical technique. The bronchial stump was carefully managed, with special consideration given to its coverage during surgery to prevent complications.

### 3.3 Enhanced educational value

This case exemplifies the intricate challenges and complexities in treating patients with multiple oncological histories, particularly when introducing targeted chemotherapy agents like Regorafenib. The rare but significant occurrence of lung cavitation leading to spontaneous pneumothorax necessitated immediate surgical intervention, while the patient's concurrent use of prednisone likely exacerbated his susceptibility to *Pseudomonas aeruginosa* pneumonia. This case serves as an educational landmark for clinicians, emphasizing the importance of vigilant monitoring and adaptability in treatment plans, particularly for those with intricate medical backgrounds.

### 3.4 Patient's perspective

While the medical intricacies of this case are undoubtedly complex, they are but one facet of the patient's life journey. Blind since childhood due to surgery for retinoblastoma, the patient has faced a multitude of challenges that extend far beyond the medical realm. Despite his visual impairment and complicated health history, he has built a fulfilling life, complete with a loving marriage and children. Throughout the diagnostic, therapeutic, and surgical interventions, what stood out was his indomitable spirit and resilience. Even when confronted with a series of severe medical complications, his optimistic outlook never wavered. He remains engaged in the fight against his multiple health challenges, buoyed by a supportive family and a strong will to continue experiencing life to its fullest. This resilience serves as a poignant reminder that the essence of medical care is not just about treating conditions but about caring for individuals in their full human context.

## 4 Methods

The work has been reported in line with the SCARE criteria [13].

## 5 Conclusion

This case report of a patient with a complex oncological history, involving childhood retinoblastoma, radiation-induced leiomyosarcoma, and skull base osteosarcoma, highlights the multifaceted challenges in cancer management, especially when employing targeted chemotherapy agents like Regorafenib. The rare but significant occurrence of lung cavitation leading to spontaneous pneumothorax in this patient underscores the necessity for vigilant monitoring and a comprehensive understanding of the potential side effects of such treatments.

The case further emphasizes the importance of considering drug-related complications in the differential diagnosis and management of acute medical conditions in oncology patients. Our patient's history of corticosteroid use, specifically prednisone, likely predisposed him to severe bacterial infection, complicating his clinical course. This aspect illustrates the need for a dynamic and adaptable treatment approach, taking into account the patient's immunocompromised status and the specific challenges posed by their comprehensive medical history.

### 5.1 Addressing surgical challenges and decisions

The surgical intervention in this case, particularly the decision to perform a lobectomy amid active infection, was marked by careful consideration of the patient's unique clinical context. The antibiotic regimen, initially chosen based on the patient's resistance profile, highlights the critical role of tailored therapeutic strategies in managing complex cases. The discussion regarding the potential use of carbapenems from the outset, considering the patient's immunocompromised state, adds an important dimension to the learning experience from this case.

### 5.2 Educational and clinical implications

This case serves as a poignant educational resource for clinicians, reinforcing the importance of a multidisciplinary approach in oncology care. It also demonstrates the critical need for continuous learning and adaptability in the face of evolving clinical scenarios. By detailing the decision-making process, surgical considerations, and management of postoperative complications, especially in the context of anti-angiogenic therapy, the report provides valuable insights for healthcare professionals dealing with similar challenging cases.

In conclusion, the management of oncology patients with complex medical histories requires not only a deep understanding of the potential complications associated with their treatment regimens but also a readiness to adapt and respond to unexpected clinical developments. This case report contributes significantly to the literature by providing a comprehensive overview of the challenges and considerations involved in such scenarios, ultimately aiming to improve patient care and outcomes.

## Consent

Written informed consent was obtained from the patient for publication of this case report and any accompanying images. A copy of the written consent is available for review on request.

## Ethical approval

Case reports are exempt from requiring ethical approval at our organization. Written informed consent is required and was obtained from the patient for publication of this case report and any accompanying images. A copy of the written consent is available for review on request.

## Funding

There is no study sponsors or other funding for this research.

## Author contribution

1- Nathaniel Grabill, MD, Primary author, Submitting and corresponding.
2- Mena Louis, DO, Co-author, corresponding

Have significantly contributed to the conceptualization, writing, and editing of this manuscript. His involvement encompasses drafting approximately half of the article and undertaking a comprehensive edit of the entire manuscript to ensure its quality and coherence.

3- Cindy Idowu, MD.

Reviewed, edited, and gave recommendations.

4- Clifton Hastings, MD

Reviewed, edited, supervised and gave recommendations.

5- Hardeep Singh, PhD

Research coordinator reviewed and edited the article.

## Guarantor

Nathaniel Grabill.

## Research registration number

This is not a “First in Man” study and has not been registered as such.

## Conflict of interest statement

All authors do not have any financial or personal relationships with other people or organizations that could inappropriately influence (bias) this work.
